# Enteral L-Arginine and Glutamine Supplementation for Prevention of NEC in Preterm Neonates

**DOI:** 10.1155/2015/856091

**Published:** 2015-03-12

**Authors:** M. S. El-Shimi, H. A. Awad, M. A. Abdelwahed, M. H. Mohamed, S. M. Khafagy, G. Saleh

**Affiliations:** Pediatric Department, Faculty of Medicine, Ain Shams University, Cairo 16088, Egypt

## Abstract

*Objective*. Evaluating the efficacy and safety of arginine and glutamine supplementation in decreasing the incidence of NEC among preterm neonates.* Methods.* Prospective case-control study done on 75 preterm neonates ≤34 weeks, divided equally into L-arginine group receiving enteral L-arginine, glutamine group receiving enteral glutamine, and control group. Serum L-arginine and glutamine levels were measured at time of enrollment (sample 1), after 14 days of enrollment (sample 2), and at time of diagnosis of NEC (sample 3).* Results*. The incidence of NEC was 9.3%. There was no difference in the frequency of NEC between L-arginine and control groups (*P* > 0.05). NEC was not detected in glutamine group; L-arginine concentrations were significantly lower in arginine group than control group in both samples while glutamine concentrations were comparable in glutamine and control groups in both samples. No significant difference was found between groups as regards number of septic episodes, duration to reach full oral intake, or duration of hospital stay.* Conclusion.* Enteral L-arginine supplementation did not seem to reduce the incidence of NEC. Enteral glutamine may have a preventive role against NEC if supplied early to preterm neonates. However, larger studies are needed to confirm these findings. This work is registered in ClinicalTrials.gov (ClinicalTrials.gov Identifier: NCT01263041).

## 1. Introduction

Necrotizing enterocolitis (NEC) is primarily a disease process of the gastrointestinal (GI) tract of premature neonates that results in inflammation and bacterial invasion of the bowel wall. Although research has presented an interesting array of potential contributing factors, the precise etiology of this multifactorial disease process remains elusive [1].

Arginine and glutamine are considered classically to be “conditionally essential” and recently are considered functionally essential amino acids [2, 3]. Arginine is the precursor for the synthesis of nitric oxide (NO) by NO synthase. Endothelial NO, an important regulator of vascular perfusion, is an anti-inflammatory chemical mediator and vasodilator involved in the maintenance of mucosal integrity, intestinal barrier function, and regulation of intestinal mucosal flow in the face of inflammation or injury. Inhibition of NO synthesis in a variety of animal models in which bowel injury is induced increases the area of intestinal damage. Recent studies reported possible value of arginine supplements for preventing severe, damaging inflammation of NEC in preterm infants [4–6].

Glutamine is essential for the growth, survival, and physiological health of actively dividing cells such as enterocytes [7]. Undergoing rapid growth, preterm infants are most likely to be exposed to severe glutamine deficiency more than term infants [8]. Recent evidence indicates that glutamine preserves the gut barrier function and prevents permeability to toxins and pathogens under various conditions of gastrointestinal mucosal injury. Glutamine is considered the most important nutrient for healing of “leaky gut syndrome” because it is the preferred fuel for enterocytes and colonocytes [9].

In this study, we hypothesized that enteral supplementation of L-arginine and glutamine may help in prevention of NEC. Thus, we aimed at evaluating the efficacy and safety of arginine and glutamine supplementation in decreasing the incidence of NEC among preterm neonates.

## 2. Subjects and Methods

### 2.1. Subjects

This prospective case-control interventional study was conducted at NICU of Maternity Hospital of Ain Shams University and NICU of Matareya Hospital, Cairo, Egypt, over a period of 26 months from January 2011 till March 2013. The study was approved by the Ethical Committee of the Pediatric Department at Ain Shams University. An informed written consent was obtained from one of the parents before enrollment of the patients. This work is registered in ClinicalTrials.gov (ClinicalTrials.gov Identifier: NCT01263041).

Neonates included in the study were 75 premature infants with gestational age ≤34 weeks assigned to receive enteral feeding within their first week of life. Newborns with congenital malformations, chromosomal abnormalities, suspected inborn error of metabolism, and sepsis, those with evidence of intraventricular hemorrhage grade ≥II on cranial ultrasound scan, with intestinal surgery, or any contraindication to early enteral feeding, were excluded from the study.

### 2.2. Methods

For all preterm neonates included in the study, gestational age was calculated based on the date of last menstrual period and confirmed by neonatal examination using the modified Ballard score [10]. Enteral feeds were commenced at 10 to 20 mL/kg per day and increased by 10 to 30 mL/kg per day as tolerated, up to a maximum of 150 to 180 mL/kg per day. Patients were fed breast milk when available, and if not, they were fed artificial milk, Bebelac premature formula (Nutricia Cuijk B.V. Holland). We do not use breast milk fortifier and we do not practice breast milk donation. Feeding intolerance was defined when residual from nasogastric tube was >30% of previous feed which resulted in decreasing or fixing the amount of feed but not stopping it [11].

Parenteral Aminoven infant (Fresenius Kabi Oncology Ltd., Austria) was started in the first 1-2 days of life at 2 g/kg per day amino acids, with increment of 0.5 gm/kg/day till reaching a total of 3.5 gm/kg/day. Parenteral amino acids are decreased gradually while increasing the enteral intake. 10% Aminoven infant contains 7.5 g of L-arginine/1000 mL. A 1-kg neonate receiving 3 gm/kg per day of a 10% Aminoven infant solution would receive 225 mg L-arginine. Breast milk contains 54 mg of L-arginine/100 mL [4].

All decisions regarding infant care including nutrition were made independently by the attending neonatologists.

Included neonates were divided into 3 groups (by simple randomization method).

- *Arginine Group.* 25 preterm newborns received enteral L-arginine with the start of enteral feedings; starting 0.75 mmol/kg/day, doubled to 1.5 mmol/kg (261 mg/kg/day) when reaching 40% of enteral feeding (60 cc/kg), till 30 days of postnatal age. Dose was divided every 12 hours and taken by oral route or via nasogastric tube [4].
- *Glutamine Group.* 25 preterm newborns received enteral glutamine with the start of feeding; starting dose of 0.156 gm/kg/day, doubled to 0.312 gm/kg/day (1 mL/kg/day) when the subject reached 40% of his enteral feeding (60 cc/kg) till 30 days of postnatal age. Dose was divided every 12 hours and taken by oral route or via nasogastric tube [12].
- *Control Group.* 25 preterm newborns started enteral feeding within first week without arginine or glutamine supplementation.

#### 2.2.1. Clinical Data

Full history was taken from mothers of neonates including maternal, obstetric, and perinatal history. Birth weights, sex, and Apgar score at 1 and 5 minutes were recorded. Complete physical examination was done with special emphasis on abdominal examination.

All preterm infants were followed up daily since the time of enrollment till they were discharged, died, or completed 30 days of life. The following were recorded:

- data related to feeding regimens (age on starting feeding, type of milk, daily increment, weight gain, evidence of feeding intolerance, and time to reach full enteral feed);
- daily measurement of weight, blood glucose level, and blood pressure;
- incidence of any stage of NEC together with surgery or death attributed to NEC; NEC was diagnosed and classified according to the criteria of Bell et al. [13];
- incidence and outcome of septic episodes; sepsis was considered when clinical deterioration occurred associated with abnormalities in total or differential leukocyte count, platelet count, and C-reactive protein with or without positive blood culture [14].

#### 2.2.2. Investigations

- Transcranial U/S was done before enrollment, before discharge, and whenever indicated.
- Abdominal X ray was done for patients suspected to have NEC.
- Assay plasma levels of L-arginine and glutamine at time of enrollment (sample 1), at day 14 of enrollment for those who did not develop NEC (sample 2), and at time of NEC diagnosis for those who developed NEC (sample 3). Venous samples were obtained one hour before feeding for enteral fed infants to avoid postprandial fluctuations [15] and at least 12 hours after any blood product transfusion. Samples were centrifuged and stored in −20°C.
  - *L-Arginine* was assayed via “Human Arginine (ARG) ELISA Kit” (Bioassay Technology Laboratory). Assay employs the quantitative sandwich enzyme immunoassay technique. Procedure was done according to manufacturer recommendations.
  - *Glutamine* was assayed using Glutamine Assay Kit (Abnova), which is based on hydrolysis of glutamine to glutamate and colorimetric determination of the product. The intensity of the product color, measured at 565 nm, is proportional to the glutamine concentration in the sample. Procedure was done according to manufacturer instructions [16].

### 2.3. Statistical Methods

#### 2.3.1. Sample Size Determination

A sample size of at least 75 cases achieved 80% power to detect a mean of paired differences of 15.0 with an estimated standard deviation of differences of 20.0 and with a significance level (alpha) 0.05 using a two-sided paired Student's *t*-test.

Data was analyzed using statistical package for special science (SPSS) software computer program version 13 (Texas, USA). Quantitative data were described using mean ± standard deviation and median (interquartile range); qualitative data were described in the form of numbers and percentages. Student's *t*-test of two independent samples was used for comparison of normally distributed quantitative variables while Mann-Whitney test was used for nonparametric data. Chi-square test was used for comparison of qualitative variables. Wilcoxon signed rank test was used to compare follow-up with initial values. The probability of error less than 0.05 was considered significant, while probabilities at 0.01 and 0.001 are highly significant.

## 3. Results

The patients' characteristics and demographic data are demonstrated in Table 1. None of our patients was exclusively breast milk fed. Five of our patients were fed both breast milk and premature formula (2 in glutamine group, 1 in arginine group, and 2 in control group). Breast milk percentage varies from nearly 100% at beginning of feeding to 30–50% close to full feeding, according to its availability. The rest of neonates used only formula milk.

The overall incidence of NEC in studied neonates was 9.3% (7 neonates); all of them were of stage II. In addition, two of our studied patients (in control group) were diagnosed as having stage I NEC but they were not regarded as having NEC due to lack of objective criteria for diagnosis. No difference in the frequency of NEC was found between L-arginine group and control group (*P* > 0.05). NEC was not detected in glutamine group with a significant statistical difference compared with the control group (*P* = 0.043). It was estimated that number needed to treat (NNT) in the glutamine group = 4.167.  Table 2 compare between the patients who developed NEC and those who did not develop NEC.

In arginine group, 2 patients were diagnosed as having NEC: one improved and one died. In control group, 5 patients were diagnosed as having NEC: one improved and 4 died. Patients who developed NEC had significantly lower gestational age and birth weight compared to those neonates who did not develop NEC. Age at diagnosis of NEC in arginine group was 10.67 ± 2.5 days, while in control group it was 9.83 ± 5.2 days (*P* > 0.05). L-Arginine level at time of diagnosis of NEC (sample 3) in arginine group was 18.6 ± 10.01 ng/mL, while sample 2 in those who did not develop NEC was 15.68 ± 3.5 ng/mL. One patient in the control group developed NEC after 14 days of enrollment; his arginine level in sample 2 = 9.4 ng/mL and in sample 3 = 12.4 ng/mL.

**Figure 1:**
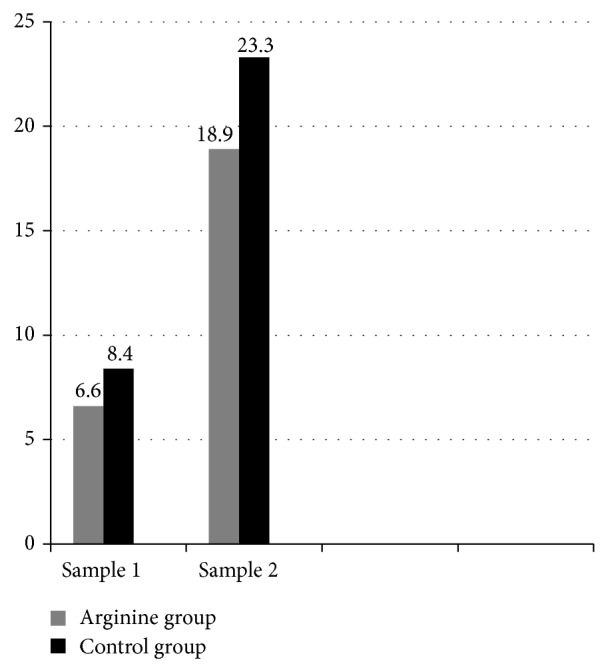
Serum arginine levels (ng/mL) in sample 1 and sample 2 in L-arginine and control groups.

L-Arginine concentrations were significantly lower in arginine group than control group at time of enrollment [median (IQ) = 6.1 ng/mL (2.4) and 8.4 ng/mL (4.3) in arginine and control groups, resp., (*P* = 0.009)] and at day 14 of enrollment [median (IQ) = 18.3 ng/mL (6.2) and 23.25 ng/mL (8.75) in arginine and control groups, resp., (*P* = 0.021)]. In each group, levels of plasma L-arginine increased significantly at day 14 compared with initial sample (*P* < 0.01) (Figure 1).

**Figure 2:**
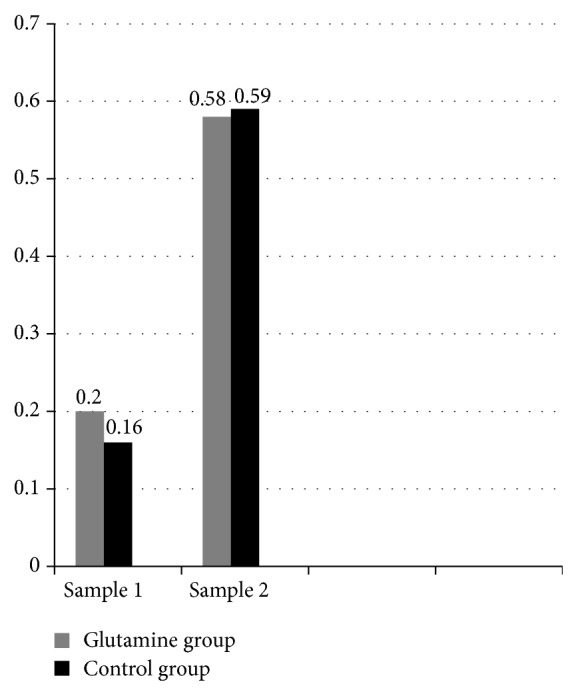
Serum glutamine levels (mM) in sample 1 and sample 2 in glutamine and control groups.

Glutamine levels were comparable in glutamine and control groups at time of enrollment [(median (IQ) = 0.2 (0.15) mM and 0.16 (0.15) mM in glutamine and control groups, resp.)] and at day 14 of enrollment [(median (IQ) = 0.58 (0.18) mM and 0.59 (0.15) mM in glutamine and control groups, resp.)]. In each group, levels of plasma glutamine increased significantly at day 14 compared with initial sample (*P* < 0.05) (Figure 2).

The increase in the levels of arginine and glutamine from sample 1 to sample 2 was comparable between arginine and control groups and between glutamine and control groups, respectively (*P* > 0.05).

There was no significant difference between studied groups as regards the number of septic episodes, duration to reach full oral intake, or duration of hospital stay. Frequencies of occurrence of hypotension or hyperglycemia were comparable between studied groups. Overall mortality among the study subjects was 13.3% (10 neonates), which was highest among control group (6 neonates) and equal in the other two groups (2 in each group). No statistical significance was found for that difference (*P* > 0.05).

## 4. Discussion

NEC is one of the most difficult diseases to eradicate, despite advances in neonatal intensive care [17]. Primary prevention of NEC should take the priority, since NEC frequently progresses from nonspecific signs, to extensive necrosis within matter of hours with medical or surgical treatment, making successful treatment and secondary prevention difficult to achieve [18].

A relative arginine deficiency or immaturity of nitric oxide synthases activity in premature infants may lead to deficient tissue NO levels, vasoconstriction, and ischemia reperfusion injury and may predispose them to NEC [19]. Plasma glutamine levels are lower in preterm infants and further decrease during acute illness, such as NEC [8].

In the current study, no significant difference was found between arginine and control groups regarding incidence of NEC or mortality from NEC. Same results were reached by others who reported that L-arginine has no role in preventing NEC [20], and a recent study assessed the preventive role of L-arginine against NEC and suggested further studies before giving recommendation for its routine use [21].

On the other hand, previous study suggested that L-arginine protects against all stages of NEC. However, inclusion of stage 1 NEC, which lacks objective criteria for identification, in the subjects is a limitation of their study. When excluding stage I, they reported that incidence of NEC was not significantly reduced by L-arginine [4]. Moreover, previous study reported that on supplementing enteral L-arginine daily, stage III of NEC, which is the most severe, was remarkably decreased [5].

In our study, L-arginine concentrations were significantly higher in control group than arginine group at time of enrollment; transplacental arginine transfer might be behind this elevation. Maternal arginine assay at time of delivery is recommended. The rise in serum arginine levels from sample 1 to sample 2 was not affected by arginine supplementation but affected by initial levels. We speculated that there might be local consumption of arginine in the GIT. However, the very few published data concerning amino acid levels in preterm neonates and the wide variability between them limited our interpretation. Reference values of arginine level varies from 95–145 *μ*mol/L in breast fed full terms [6] to 19 *μ*mol/L in VLBW infants (<29 weeks) at day 3 of life [22]. An interesting finding in the current study was that the serum level of L-arginine was generally low in all the samples if compared to levels in the literature. We did not find a study done in Egypt that measured L-arginine in preterms to refer to, as that may hypothesize the possible racial or ethnic difference that may be the cause for that finding.

In the current study, no detected cases of NEC were found in glutamine group. Similarly, previous studies reported that parenteral glutamine supplementation from day 4 to day 14 among VLBW neonates decreased the incidence of NEC [23, 24]. In addition, others concluded that enteral glutamine may have a beneficial role in intestinal integrity and the overall incidence of NEC/septicemia in preterm infants [25]. Enteral glutamine supplementation can stabilize the intestinal barrier by preventing translocation of bacteria or other toxins and ameliorating the subsequent induction of a systemic inflammatory response. Glutamine will stabilize intestinal barrier function via promotion of intercellular junction integrity, provision of protective mucus, and providing the ability for new cells to proliferate after injury. In addition, glutamine produces glutathione which plays a role in preventing oxidative damage in the intestine [26]. On the other hand, others reported that enteral supplementation of glutamine had no effect on the incidence of necrotizing enterocolitis in VLBW neonates [27]. Glutamine levels withdrawn before supplementation were comparable between NEC positive and NEC negative groups. We speculate that serum glutamine levels do not reflect the splanchnic levels.

We did not find an effect for glutamine supplementation on serum glutamine levels which could be explained by previous studies in healthy adults which showed that over 60% of the enteral glutamine undergoes first-pass extraction by the splanchnic compartment [28]. Moreover, a study reported that enteral supplementation of glutamine seems to be locally used by the intestine but may not help entering the systemic circulation to enhance the immune response [3]. In all our patients even in control group, there is an increase in serum levels of arginine and glutamine in sample 2 compared with sample 1 which suggest postnatal increments in the levels of these 2 amino acids.

As regards safety of enteral supplementation of L arginine, alteration in blood sugar, hypotension, and bleeding tendency (e.g., intracranial hemorrhage) were the most feared complications. All neonates involved in the study tolerated well the received supplementations without any noticeable increase in incidence of hypotension or hyperglycemia. Similarly, previous studies reported no side effects of arginine supplementation to VLBW neonates [5, 6], while others concluded that arginine supplementation in septic patients has transient effects on hemodynamics when supplied as a bolus but seems without hemodynamic side effects when supplied continuously as it could have an essential role in combating infection and sepsis. However, still, the use of L-arginine in sepsis is a matter of debate [29].

In the current study, no additional benefit was observed in the glutamine group regarding frequency of septic episodes or duration of hospital stay compared to control group. Similarly, previous studies reported that glutamine supplementation in VLBW did not influence the incidence of hospital acquired sepsis, as well as the length of stay in the intensive care unit, and the total time of hospitalization [24, 26, 30]. Alternatively, others conducted a double blind placebo controlled study and reported that infectious morbidity was significantly lowered in infants who received glutamine-enriched enteral nutrition [12].

In conclusion, enteral L-arginine supplementation can be safely administrated, with a dose of 1.5 mmol/kg/d, to preterm neonates for duration of about 28 days. Enteral L-arginine supplementation did not seem to reduce the incidence of NEC. Enteral glutamine may have a preventive role against NEC if supplied early to preterm neonates. However, larger studies are needed to confirm these findings.

## Figures and Tables

**Table 1:** Clinical characteristics of the 3 studied groups.

	L-Arginine group *n* = 25	Glutamine group *n* = 25	Control group *n* = 25	*Z*/*χ* ^2^	*P* value
GA (weeks)	27–34 31.84 ± 2.29	28–34 31.68 ± 1.35	26–34 30.64 ± 2.34	*Z*: −1.777	0.76
BW (kg)	0.97–1.55 1.45 ± 0.26	1.16–1.55 1.45 ± 0.21	0.9–1.6 1.31 ± 0.25	*Z*: −2.02	**0.043** ^*^
Age of start of feeding (days)	2.2 ± 1.443	3.68 ± 1.35	1.79 ± 0.79	*Z*: −0.89	0.37

	Median (IQ)	Median (IQ)	Median (IQ)		

Apgar					
(1 min)	6 (2)	5 (2)	7 (2)	*Z*: −1.34	0.18
(5 min)	8 (2)	8 (1.5)	8 (2)	*Z*: −0.15	0.88

	*N* (%)	*N* (%)	*N* (%)		

Gender					
Male	11 (44%)	12 (48%)	11 (44%)	*χ* ^2^: 0.11	0.95
Female	14 (56%)	13 (52%)	14 (56%)		
MOD					
CS	18 (72%)	23 (92%)	22 (88%)	*χ* ^2^: 6.11	0.19
Vaginal	7 (28%)	2 (8%)	3 (12%)		
Prolonged PROM	9 (36%)	4 (16%)	4 (16%)	*χ* ^2^: 3.804	0.149
PDA	5 (20)	6 (24%)	8 (32%)	*χ* ^2^: 0.987	0.61
Inotropes	6 (24%)	10 (40%)	8 (32%)	*χ* ^2^: 1.47	0.479
CLD	3 (12%)	2 (8%)	0 (0%)	*χ* ^2^: 3	0.223
SGA	12 (48%)	11 (44%)	7 (28%)	*χ* ^2^: 2.33	0.311

GA: gestational age, BW: birth weight, kg: kilogram, CS: caesarean section, MOD: mode of delivery, PROM: premature rupture of membrane, PDA: Patent ductus arteriosus, and CLD: chronic lung disease.

SGA: small for gestational age, ^*^significant.

*Z*: Mann-Whitney test, *χ* ^2^: chi square.

**Table 2:** Comparison between NEC negative and NEC positive patients.

	NEC negative	NEC positive	*P*	Odds ratio	95% CI
	Number = 68	Number = 7			
GA (mean ± SD)	31.71 ± 2.05	28.86 ± 2.04	**0.001** ^*^	0.530	0.3405 to 0.8240
WT (mean ± SD)	1.43 ± 0.23	1.11 ± 0.18	**0.001** ^*^	0.000	0.0000 to 0.0758
Age on start (mean ± SD)	2.67 ± 1.52	1.57 ± 0.79	0.064	0.461	0.1896 to 1.1182
Increment of milk [number (%)]					
≤20 cc/kg/day (*n*: 64)	58 (85.3%)	6 (85.7%)	0.976	0.967	0.1049 to 8.9075
>20 cc/kg/day (*n*: 11)	10 (14.7%)	1 (14.3%)			
MOD [number (%)]					
NVD	10 (14.7%)	2 (28.6%)	0.352	0.431	0.0733 to 2.5353
CS	58 (85.3%)	5 (71.4%)			
Gender [number (%)]					
Female	37 (54.4%)	4 (57.1%)	0.890	0.895	0.1860 to 4.3079
Male	31 (45.6%)	3 (42.9%)			
Arginine 1 (ng/mL) (mean ± SD)	8.12 ± 2.78	8.93 ± 4.28	0.497	1.090	0.8523 to 1.3934
Glutamine 1 (mM) (mean ± SD)	0.20 ± 0.10	0.23 ± 0.20	0.481	1.110	0.1754 to 1.231

GA: gestational age. WT: weight. MOD: mode of delivery. NVD: normal vaginal delivery.

CS: Caesarean section. 1: at time of enrollment. ^*^Highly significant.

## Abbreviations

NEC: Necrotizing enterocolitis
NICU: Neonatal intensive care unit.
